# Variants of lipopeptides and glycolipids produced by *Bacillus amyloliquefaciens* and *Pseudomonas aeruginosa* cultured in different carbon substrates

**DOI:** 10.1186/s13568-017-0367-4

**Published:** 2017-05-31

**Authors:** Thando Ndlovu, Marina Rautenbach, Sehaam Khan, Wesaal Khan

**Affiliations:** 1 Department of Microbiology, Faculty of Science, Stellenbosch University, Private Bag X1, Stellenbosch, 7602 South Africa; 2 BIOPEP Peptide Group, Department of Biochemistry, Faculty of Science, Stellenbosch University, Private Bag X1, Stellenbosch, 7602 South Africa; 3 Faculty of Health and Applied Sciences, Namibia University of Science and Technology, 13 Storch Street, Private Bag 13388, Windhoek, Namibia

**Keywords:** Surfactin, Rhamnolipid, *Bacillus amyloliquefaciens* ST34, *Pseudomonas aeruginosa* ST5, Carbon sources, UPLC–MS

## Abstract

The quantitative and qualitative effect of water immiscible and miscible carbon-rich substrates on the production of biosurfactants, surfactin and rhamnolipids, by *Bacillus amyloliquefaciens* ST34 and *Pseudomonas aeruginosa* ST5, respectively, was analysed. A small-scale high throughput 96 deep-well micro-culture method was utilised to cultivate the two strains in mineral salt medium (MSM) supplemented with the water miscible (glucose, glycerol, fructose and sucrose) and water immiscible carbon sources (diesel, kerosene and sunflower oil) under the same growth conditions. The biosurfactants produced by the two strains were isolated by acid precipitation followed by an organic solvent extraction. Ultra-performance liquid chromatography coupled to electrospray ionisation mass spectrometry was utilised to analyse yields and characterise the biosurfactant variants. For *B. amyloliquefaciens* ST34, maximum surfactin production was observed in the MSM supplemented with fructose (28 mg L^−1^). In addition, four surfactin analogues were produced by ST34 using the different substrates, however, the C_13_–C_15_ surfactins were dominant in all extracts. For *P. aeruginosa* ST5, maximum rhamnolipid production was observed in the MSM supplemented with glucose (307 mg L^−1^). In addition, six rhamnolipid congeners were produced by ST5 using different substrates, however, Rha–Rha–C_10_–C_10_ and Rha–C_10_–C_10_ were the most abundant in all extracts. This study highlights that the carbon sources utilised influences the yield and analogues/congeners of surfactin and rhamnolipids produced by *B. amyloliquefaciens* and *P. aeruginosa*, respectively. Additionally, glucose and fructose were suitable substrates for rhamnolipid and surfactin, produced by *P. aeruginosa* ST5 and *B. amyloliquefaciens* ST34, which can be exploited for bioremediation or as antimicrobial agents.

**Electronic supplementary material:**

The online version of this article (doi:10.1186/s13568-017-0367-4) contains supplementary material, which is available to authorized users.

## Introduction

Biosurfactants are an important class of microbially synthesised compounds that have been extensively researched due to their diverse biological properties and functions (Van Hamme et al. 2006; Gudiña et al. 2013; Kiran et al. 2016). Moreover, owing to their low toxicity and biodegradable nature, they exhibit potential for various commercial applications as environmentally friendly alternatives to synthetic surfactants (Nitschke and Costa 2007). Lipopeptides and glycolipids, in particular, have been exploited for their potential to serve as antimicrobial, antiadhesive, antitumor and antizoospore agents in the medical and pharmaceutical industries (Banat et al. 2010; Raaijmakers et al. 2010).

Lipopeptides are synthesised by means of a multistep pathway mediated by various non-ribosomal peptide synthetase (NRPS) enzymes which catalyse the condensation and selection of amino acid residues to yield various metabolites. Gene expression for surfactin production in *Bacillus* species is reported to be cell density dependent and occurs predominantly in the late exponential and stationary phases of bacterial growth (Gross and Loper 2009). Structural diversity of the lipopeptides then ranges from the varying composition and length of the hydrophobic moiety to the type, number and the configuration of the amino acid present in the hydrophilic moiety (Roongsawang et al. 2010). The lipopeptide structural diversity can significantly influence their biological and physicochemical properties (Bonmatin et al. 2003; Das et al. 2009; Singh et al. 2014), however, lipopeptides are not generally utilised for large-scale commercial production due to the high costs (substrates and downstream processes) associated with their production.

The most effective glycolipids, with strong emulsification and surface activities as well as antimicrobial and antiadhesive properties are rhamnolipids. They are primarily produced by *Pseudomonas aeruginosa* strains as the most prominent secondary metabolite (Syldatk et al. 1985). Rhamnolipid biosynthesis by *P. aeruginosa* occurs in consecutive steps of glycosyl transfer reactions catalysed by different rhamnosyl-transferases, yielding separate activated precursor hydrophilic (mono- or dirhamnose) and hydrophobic (hydoxyfatty acids) moieties. These are then dimerised by the rhamnosyl-transferases and other enzymes (Soberón–Chávez et al. 2005). The production of rhamnolipids by *P. aeruginosa* is tightly regulated by a quorum sensing mechanism, in response to both environmental stress and nutritional factors (Deziel et al. 2003; Reis et al. 2011; Geys et al. 2014). The microbially produced rhamnolipid mixtures display varying properties that depend on the type and proportion of the homologs, which differ, based on the bacterial strain used, culture conditions, medium composition and the type of carbon source used for growth (Déziel et al. 1999; Abalos et al. 2001; Das et al. 2009; Singh et al. 2014).

The selection of a cost-effective substrate to produce biosurfactants is thus particularly crucial for large-scale production, as different types of carbon sources are reported to markedly influence the concentration of biosurfactant compounds produced. In addition, relevant published research has emphasised on the effect of a carbon source has on the specific congeners/homologues of biosurfactants synthesised by a specific microbial strain (Bonmatin et al. 2003; Das et al. 2009; Singh et al. 2014). A study conducted by Kim et al. (1997) assessed the use of emulsified n-hexadecane, soybean oil and glucose to produce a lipopeptide biosurfactant using a *B. subtilis* C9 strain. Results indicated that the lipopeptide biosurfactant was produced only when glucose was used as a carbon source. Thaniyavarn et al. (2006) also investigated the production of biosurfactants using *P. aeruginosa* A41 isolated from seawater. The microbe was cultured either in a vegetable oil (olive, palm and coconut oils) or a fatty acid (lauric, myristic, palmitic, stearic, oleic or linoleic acids) as the main carbon source. Different rhamnolipid concentrations of 2.91, 2.93 and 6.58 g L^−1^ were obtained with the palm, coconut and olive oils, respectively. In the case of the fatty acid substrates, the rhamnolipid concentration ranged from 0.26 g L^−1^ (palmitic acid) to 4.99 g L^−1^ (linoleic acid). However, the rhamnolipid obtained when *P. aeruginosa* UW-1 was cultured in fatty acids had shorter chain lengths and caused a high oil displacement activity when compared with yields obtained when vegetable oil was used (Thaniyavarn et al. 2006). The authors then concluded that cost-effective production of industrial volumes of rhamnolipid was possible when using *P. aeruginosa* UW-1 isolates cultured using palm oil as the carbon source.

The primary aim of the current study was to assess the quantitative and qualitative effects of different carbon sources have on the production of rhamnolipid and surfactin by *Pseudomonas aeruginosa* ST5 and *Bacillus amyloliquefaciens* ST34, respectively. This objective was achieved by culturing each bacterial strain on mineral salt medium (MSM) supplemented with water miscible (glucose, glycerol, fructose and sucrose) or water immiscible carbon substrates (diesel, kerosene and sunflower oil) using the high throughput production method as previously described by Vosloo et al. (2013). Ultra-performance liquid chromatography coupled to electrospray ionisation mass spectrometry (UPLC–ESI-MS) was then used to characterise the crude biosurfactant compounds and determine their respective approximate concentrations. The ideal carbon sources required by each microorganism for maximum yields and diversity of biosurfactant compounds were identified.

## Materials and methods

### Pre-culturing of biosurfactant producing isolates

Biosurfactant producing bacteria were isolated from wastewater samples collected from Stellenbosch wastewater treatment plant in the Western Cape, South Africa (GPS co-ordinates: −33.943505, 18.824584) as described by Ndlovu et al. (2016). The bacterial isolates ST34, identified as *B. amyloliquefaciens* (collection number SARCC 696 at the South African Rhizobium Culture Collection) and ST5, identified as *P. aeruginosa* (collection number SARCC 697 at the South African Rhizobium Culture Collection), using molecular characterisation (Ndlovu et al. 2016), were utilised in the current study. Henceforth the *B.* *amyloliquefaciens* and *P. aeruginosa* isolates will be referred to by their code identifiers, ST34 and ST5, respectively. Utilising a UPLC–MS method, the ST34 and ST5 strains have previously been shown to produce surfactin and rhamnolipid biosurfactants, respectively (Ndlovu 2017). The ST34 and ST5 bacterial strains were thus utilised in the current study to assess the effect of MSM supplemented with various water immiscible and miscible substrates as sole carbon sources for the production of various surfactin analogues and rhamnolipid congeners.

The bacterial isolates were maintained in 40% glycerol at −80 °C. A loopful of the glycerol stock of each isolate was transferred onto nutrient agar, streaked and incubated at 37 °C for 18–24 h. Single colonies were inoculated onto 5 mL Luria–Bertani (LB) broth, and incubated at 37 °C for 18–24 h. This inoculum was used as a seed culture to inoculate the MSM that was supplemented with various carbon sources (diesel, fructose, glucose, glycerol, kerosene, sucrose and sunflower oil).

### High throughput 96 deep-well production of biosurfactants

The high throughput 96 deep-well micro-culture production method was adapted from a previous study conducted by Vosloo et al. (2013). Mineral salt medium was prepared as previously described by Silva et al. (2010) and was supplemented with various substrates as sole carbon sources as follows: 3% diesel (Total South Africa, Johannesburg, South Africa), 3% d(−) fructose (Saarchem (Pty) LTD, Johannesburg, South Africa), 3% d(+) monohydrate glucose (Kimix chemicals and lab suppliers cc, Cape Town, South Africa), 3% glycerol (Merck, Darmstadt, Germany), 3% kerosene (Sigma-Aldrich, St. Louis, USA), 3% sucrose (Merck chemicals, Johannesburg, South Africa) and 3% sunflower oil (SPAR South Africa (Pty) LTD, Pinetown, South Africa). Aliquots of 50 µL of the overnight culture broth of each bacterium (ST34 or ST5) were then pipetted into the wells (in triplicate) of the sterile 96 deep-well plate containing 500 µL of MSM supplemented with 3% of the respective substrates utilised as sole carbon sources. The 96 deep-well plates were sealed and were incubated for 120 h at 30 °C on an orbital shaker (MRCLAB, London, UK) (Vosloo et al. 2013).

The solvent extraction of biosurfactant compounds produced by ST34 and ST5 was conducted as outlined in Vosloo et al. (2013). The ST34 and ST5 strains cultured in the respective carbon sources in the 96 deep-well plate were acidified with concentrated hydrochloric acid (HCl, Merck, Darmstadt, Germany) to a pH of approximately 4 and were allowed to stand at ambient temperature for 24 h. Thereafter, the 96 deep-well plates were centrifuged at 2200×*g* for 60 min, the pellets were re-suspended in 200 µL of 100% acetonitrile (Romil, Cambridge, UK) and were sonicated for 15 min. A further 200 µL of analytical quality water (prepared through a MilliQ system from Millipore, Billerica, USA) was added to each well, the plates were sonicated for 15 min and then centrifuged at 2200×*g* for 30 min. Respective supernatants were then transferred into analytically weighed vials, lyophilised and the mass for each extract was analytically determined. Extracts were then dissolved in 70% (*v/v*) acetonitrile to 10.00 mg mL^−1^, centrifuged at 8600×*g* for 10 min to remove particulates and a ten times dilution was performed using analytical quality water to obtain a final concentration of 1.00 mg mL^−1^. Extracts were subsequently analysed using UPLC–MS coupled to electrospray ionisation mass spectrometry (ESI).

### Analysis with ultra-performance liquid chromatography linked to mass spectrometry

Electrospray ionisation mass spectrometry analyses were conducted in the LCMS Central Analytical Facility at Stellenbosch University. A Waters Quadrupole Time-of-Flight Synapt G2 (Waters Corporation, Milford, USA) mass spectrometer was utilised for the ESI-MS and was coupled to an Acquity UPLC for the UPLC–MS analysis of the biosurfactant extracts. All extracts were subjected to UPLC–MS analysis. Briefly, 3 µL sample (each extract obtained from MSM supplemented with different substrates as sole carbon sources) was separated on an Acquity UPLC C18 reverse-phase analytical column (Acquity UPLC^®^ HSS T3, 1.8 µm particle size, 2.1 × 150 mm, Waters corporation, Dublin, Ireland) at a flow rate of 0.300 mL min^−1^ using a 0.1% formic acid (A) to acetonitrile (B) gradient [60% (A) from 0 to 0.5 min for loading, gradient was from 40 to 95% (B) from 0.5 to 11 min and then 95 to 40% (B) from 15 to 18 min]. The UPLC–ESI-MS profiles of the biosurfactant compounds were compared to those obtained for surfactin and rhamnolipid standards (Sigma-Aldrich, St. Louis, USA). The approximate yields of the surfactin and rhamnolipid compounds in the solvent extracts obtained from the ST34 and ST5 cultures, respectively, were also determined using the surfactin and rhamnolipid standards (concentration of 1.00 mg mL^−1^).

The analytes were subjected to a capillary voltage of 3 kV, cone voltage of 15 V and a source temperature of 120 °C. Data acquisition in the positive mode was performed by MS scanning a second analyser through the *m/z* range of 200–3000 and the data was thereafter analysed using MassLynx software version 4.1 SCN 714 (Waters Corporation, Milford, USA).

### Statistical analysis

The yield of surfactin and rhamnolipids produced by ST34 and ST5 strains, respectively, grown in the different substrates were expressed as mean values ± standard error of mean. The one-way analysis of variance (ANOVA) was then utilised to determine the statistical difference in the yield of surfactin and rhamnolipids produced by ST34 and ST5, respectively, when grown on various substrates as sole carbon sources. GraphPad Prism software version 7.02 (GraphPad Software, Inc. San Diego, USA) was utilised to perform one-way ANOVA. The data was considered statistically significant if p < 0.05.

## Results

The *B. amyloliquefaciens* ST34 and *P. aeruginosa* ST5 strains utilised in the current study, were previously shown to produce surfactin and rhamnolipids, respectively when cultivated in MSM supplemented with glycerol as a sole carbon source (Ndlovu 2017). In the current study, the production profile of surfactin and rhamnolipids by the ST34 and ST5 strains, respectively, when cultured in MSM supplemented with a variety of alternative carbon sources was assessed.

### Effect of carbon source on the surfactin production by *Bacillus amyloliquefaciens* ST34

A small-scale high throughput micro-culture method (96 deep-well plate) was utilised to culture the ST34 strain in MSM supplemented with different water immiscible (diesel, kerosene and sunflower oil) and water miscible (glycerol, glucose, fructose and sucrose) substrates (Vosloo et al. 2013). The extracts were obtained from the ST34 MSM cultures (96 deep-well plates) by the solvent (acetonitrile) extraction method and were subjected to UPLC–MS analysis.

**Fig. 1:**
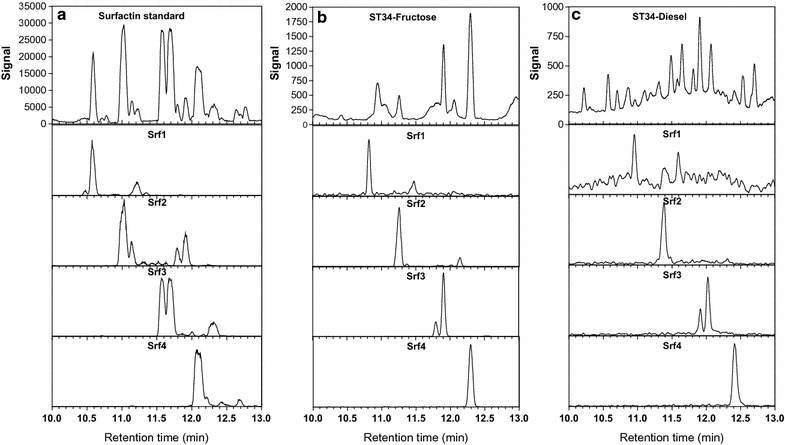
UPLC–MS profiles of the surfactin standard (**a**); ST34 Fructose-MSM extract (**b**); ST34 Diesel-MSM extract (**c**). The *top row* profiles depict the signal of positive molecular ions detected between 10 and 13 min. Note the difference in* Y axis* which are a direct indication of amounts. The profiles below *each top row* spectrum show the extracted spectra of the four surfactin groups with Srf1 = *m/z* 994.65, Srf2 = *m/z* 1008.66; Srf3 = *m/z* 1022.68 and Srf4 = *m/z* 1036

For all the ST34 extracts (obtained from MSM supplemented with different carbon-rich substrates), the ion spectra in positive mode showed the main surfactins with molecular ions at *m/z* 1008.66, 1022.68 and 1036.69, which corresponded to the protonated singly charged species [M+H]^+^ (Additional file 1: Figures S1, S2; Table 1). The ion spectra in positive mode also showed the minor surfactin group at *m/z* 994.65 (results not shown). Within the spectrum, singly charged protonated molecular species [M+H]^+^ of each type of surfactin differed by a mass of 21.99 atomic mass units (amu) and this difference was consistent with the expected singly charged sodiated molecules [M+Na]^+^ observed at *m/z* 1016.63, 1030.64, 1044.66 and 1058.68 (Table 1). The observed relative molecular mass (*M*_r_) values of the four groups of molecules denoted Srf1–4, corresponded to the expected *M*_r_ values of known surfactin analogues (Additional file 1: Figures S1, S2; Table 1). The UPLC–MS profiles of the surfactin standard and the extracts produced by ST34 cultivated in MSM supplemented with the water miscible substrates (glucose, fructose, sucrose and glycerol) and water immiscible substrates (diesel, kerosene and sunflower oil) revealed four major peaks/peak clusters with retention times (R_t_) between 10 and 13 min (Fig. 1). In the current study the surfactin groups then eluted as follows, surfactin group 1 (Srf1) (R_t_ 10.7, 10.8, 11.5, 11.6 min), Srf2 (R_t_ 11.3, 11.4, 12.1, 12.2 min), Srf3 (R_t_ 11.8, 11.9, 12 min) and Srf4 (R_t_ 12.4 min) (Table 1; Fig. 1; Additional file 1: Figures S1, S2).

**Table 1:** Summary of the surfactins extracted from *B. amyloliquefaciens* ST34, as detected with high resolution mass spectrometry (<10 ppm)

Surfactin group (Abbr)	R_t_ (min)^a^	Characterised and proposed* peptide sequences in surfactin group	Mono-isotopic Exp/Theor *M*_r_	Protonated species Exp/Theor *m/z*	Sodiated species Exp/Theor*m/z*
Surfactin 1 (Srf1)	10.7; 10.8; 11.5; 11.6	Cyclo[(**C**_**13**_ **H**_**24**_ **O**_**2**_)-l-Glu-**l** **-Leu**-d-Leu-l-Val-l-Asp-l-Leu-**l** **-Val**]	993.6376	994.6473	1016.6365
		Cyclo[(**C**_**13**_ **H**_**24**_ **O**_**2**_)-l-Glu-**l** **-Ile**-D-Leu-l-Val-l-Asp-l-Leu-**l** **-Val**]	993.6403	994.6481	1016.6259
Surfactin 2 (Srf2)	11.3; 11.4; 12.1; 12.2	Cyclo[(**C**_**14**_ **H**_**26**_ **O**_**2**_)-l-Glu-**l** **-Leu**-D-Leu-l-Val-l-Asp-l-Leu-**l** **-Val**] Cyclo[(**C**_**14**_ **H**_**26**_ **O**_**2**_)-l-Glu-**l** **-Ile**-D-Leu-l-Val-l-Asp-l-Leu-**l** **-Val**]	1007.6565	1008.6608	1030.6415
		Cyclo-[(**C**_**13**_ **H**_**24**_ **O**_**2**_)-l-Glu-**l** **-Leu**-D-Leu-l-Val-l-Asp-l-Leu-**l** **-Leu**] Cyclo[(**C**_**13**_ **H**_**24**_ **O**_**2**_)-l-Glu-**l** **-Leu**-D-Leu-l-Val-l-Asp-l-Leu-**l** **-Ile**] *Cyclo-[(**C**_**13**_ **H**_**24**_ **O**_2_)-l-Glu-**l** **-Ile**-D-Leu-l-Val-l-Asp-l-Leu-**l** **-Leu**] *Cyclo-[(**C**_**13**_ **H**_**24**_ **O**_**2**_)-l-Glu-**l** **-Ile**-D-Leu-l-Val-l-Asp-l-Leu-**l** **-Ile**]			
			1007.6552	1008.6596	1030.6416
Surfactin 3 (Srf3)	11.8; 11.9; 12.0	Cyclo[(**C**_**15**_ **H**_**28**_ **O**_**2**_)-l-Glu-**l** **-Leu**-D-Leu-l-Val-l-Asp-l-Leu-**l** **-Val**] Cyclo[(**C**_**15**_ **H**_**28**_ **O**_**2**_)-l-Glu-**l** **-Ile**-D-Leu-l-Val-l-Asp-l-Leu-**l** **-Val**]	1021.6693	1022.6773	1044.6582
		Cyclo[(**C**_**14**_ **H**_**26**_ **O**_**2**_)-l-Glu-**l** **-Leu**-D-Leu-l-Val-l-Asp-l-Leu-**l** **-Leu**] cyclo[(**C**_**14**_ **H**_**26**_ **O**_**2**_)-l-Glu-**l** **-Leu**-D-Leu-l-Val-l-Asp-l-Leu-**l** **-Ile**] *Cyclo-[(**C**_**14**_ **H**_**26**_ **O**_**2**_)-l-Glu-**l** **-Ile**-D-Leu-l-Val-l-Asp-l-Leu-**l** **-Leu**] *Cyclo-[(**C**_**14**_ **H**_**26**_ **O**_**2**_)-l-Glu-**l** **-Ile**-D-Leu-l-Val-l-Asp-l-Leu-**l** **-Ile**]			
			1021.6715	1022.6752	1044.6572
Surfactin 4 (Srf4)	12.4	Cyclo[(**C**_**15**_ **H**_**28**_ **O**_**2**_)-l-Glu-**l** **-Leu**-D-Leu-l-Val-l-Asp-l-Leu-**l** **-Leu**] Cyclo[(**C**_**15**_ **H**_**28**_ **O**_**2**_)-l-Glu-**l** **-Leu**-D-Leu-l-Val-l-Asp-l-Leu-**l** **-Ile**] *Cyclo[(**C**_**15**_ **H**_**28**_ **O**_**2**_)-l-Glu-**l** **-Ile**-D-Leu-l-Val-l-Asp-l-Leu-**l** **-Leu**] Cyclo[(**C**_**15**_ **H**_**28**_ **O**_**2**_)-l-Glu-**l** **-Ile**-D-Leu-l-Val-l-Asp-l-Leu-**l** **-Ile**]	1035.6819	1036.6896	1058.6737
			1035.6881	1036.6909	1058.6729

The proposed chemical structures, theoretical (Theor) and experimental (Exp) *M*_r_ and monoisotopic *m/z* values, as well as observed UPLC retention times for representative examples are provided

^a^UPLC retention time of main peaks corresponding to the groups *m/z* values

### Relative quantification of surfactin in ST34 extracts

The approximate yields of the surfactin compounds in the solvent extracts obtained from the ST34 cultures were determined using the surfactin standard. This was achieved by totalling the ionisation intensities of all the protonated [M+H]^+^ surfactin groups (Srf1–4) detected in standard surfactin (Table 2), which was assumed equal to 1.00 mg mL^−1^ for comparative purposes only, as the absolute purity of the surfactin standard is unknown. The signal intensity of each surfactin group was then utilised to determine the concentration of the respective individual surfactin groups in the ST34 extracts relative to that in the characterised standard surfactin (Table 2). The ST34 extracts were divided into two groups based on the different type of substrate (water miscible and immiscible) utilised as a source of carbon for the growth and production of surfactin by the ST34 strain. For the water immiscible substrates (diesel, sunflower oil and kerosene), the ST34 strain produced the highest total surfactin of 6.0 ± 1.6 mg L^−1^ in the extract obtained from the MSM supplemented with diesel, while the lowest concentration of 3.3 ± 1.9 mg L^−1^ was observed in the MSM supplemented with sunflower oil (Table 2).

**Table 2:** Comparison of the approximate quantities of each surfactin group and the total surfactin production profile by *B. amyloliquefaciens* ST34 grown in mineral salt medium supplemented with different substrates as sole carbon sources

Carbon substrate	Surfactin groups (mg L^−1^)				Total surfactin mg L^−1^ culture
	Srf1	Srf2	Srf3	Srf4	
Diesel	0.8 ± 0.2	1.4 ± 0.3	1.5 ± 0.4	2.3 ± 0.7	6.0 ± 1.6
Kerosene	0.3 ± 0.1	0.9 ± 0.5	1.0 ± 0.6	1.8 ± 1.2	4.1 ± 2.3
Sunflower oil	0.3 ± 0.04	0.7 ± 0.3	0.9 ± 0.5	1.4 ± 1.0	3.3 ± 1.9
Fructose	1.1 ± 0.7	4.4 ± 3.0	11.2 ± 8.6	11 ± 3.9	28 ± 16
Glucose	0.4 ± 0.1	0.7 ± 0.3	1.5 ± 0.9	1.2 ± 0.6	3.7 ± 1.9
Glycerol	0.4 ± 0.1	1.1 ± 0.3	1.2 ± 0.4	1.6 ± 0.4	4.3 ± 1.2
Sucrose	0.5 ± 0.1	1.3 ± 0.1	3.4 ± 0.9	2.3 ± 1.0	7.6 ± 2.0
Surfactin standard	215.09	400.82	318.45	58.74	1000^a^

Each value represents the average of three culture extracts with standard error of the mean (SEM)

^a^Total concentration of standard surfactin include concentration of the other surfactin variants observed at 6.9 mg L^−1^

**Fig. 2:**
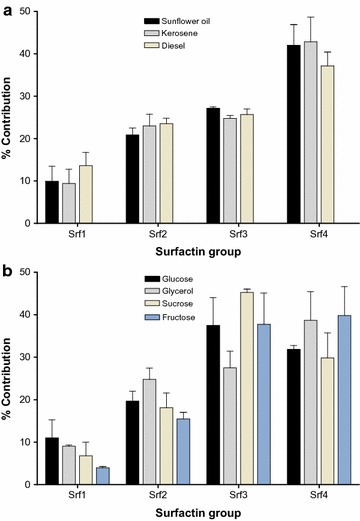
Comparison of the extracts obtained from ST34 cultivated in mineral salt medium supplemented with **a** water immiscible substrates and **b** water miscible substrates, showing the relative contribution of each of the surfactin groups in the biosurfactant extracts. The contribution was calculated from UPLC profiles, with the assumption that all the surfactin species have similar ion responses. *Each bar* represents the average of three culture extracts with standard error of the mean (SEM)

The relative contribution for each surfactin group in an extract is illustrated in Fig. 2a, which indicated that the Srf1 group was below 15% abundance in all three ST34 extracts obtained from the water immiscible substrates. The Srf2, Srf3 and Srf4 were the main surfactin groups detected in the ST34 extracts obtained for the water immiscible substrates as illustrated in Fig. 2a. The Srf4 group containing a longer branched fatty acyl chain (C_15_), was produced in higher quantities, with a relative abundance of 37, 42 and 43% in the ST34 extracts obtained from the diesel, sunflower oil and the kerosene, respectively (Fig. 2a). The total surfactin concentration of the Srf4 group then corresponded to 2.3 ± 0.7, 1.4 ± 1.0 and 1.8 ± 1.2 mg L^−1^, in the ST34 extracts obtained from the MSM supplemented with diesel, sunflower oil and kerosene, respectively (Table 2).

For the water miscible substrates (glucose, glycerol, fructose and sucrose), the ST34 strain produced the highest total surfactin of 28 ± 16 mg L^−1^ in the extract obtained from the MSM supplemented with fructose, while the lowest concentration of 3.7 ± 1.9 mg L^−1^ was obtained in the MSM supplemented with glucose (Table 2). The surfactin Srf1 group constituted approximately 11% relative abundance of the total surfactin produced by the ST34 strain grown in water miscible substrates (glucose, glycerol, fructose and sucrose) (Fig. 2b). Similar to the results obtained for the water immiscible substrates, Srf2, Srf3 and Srf4 were the main surfactin groups detected in the ST34 extracts obtained in the water miscible substrates as illustrated in Fig. 2b. The highest relative abundance of the Srf2 group (25%) was obtained in the glycerol extract, while the fructose extract yielded a 16% relative abundance (Fig. 2b; Table 2). For the Srf3 group, the highest relative abundance of 44% was observed in the sucrose extract, while the lowest abundance of 28% was observed in the glycerol extract. The Srf4 group then showed a relative abundance of 39, 37, 32, and 30% in the ST34 extracts obtained from the fructose, glycerol, glucose and sucrose, respectively (Fig. 2b). The total surfactin concentration of the Srf4 group then corresponded to 11 ± 3.9, 1.6 ± 0.4, 1.2 ± 0.6 and 2.3 ± 1.0 mg L^−1^, in the ST34 extracts obtained from the MSM supplemented with fructose, glycerol, glucose and sucrose, respectively (Table 2).

Statistical analysis was performed to determine if there was any significant difference between the surfactin yields when ST34 was grown in MSM supplemented with the different substrates. ANOVA analysis then indicated that no significant difference was observed between the surfactin quantities produced by the ST34 cultivated in MSM supplemented with water immiscible substrates [diesel vs kerosene (p = 0.99), diesel vs sunflower (p = 0.95) and kerosene vs sunflower oil (p > 0.99)]. For the water miscible substrates, ANOVA analysis also indicated no significant difference in the quantities of surfactin produced by ST34 grown in glucose, glycerol and sucrose [glucose vs glycerol (p > 0.99), glucose vs sucrose (p = 0.89) and glycerol vs sucrose (p = 0.95)]. However, a significant difference in the concentration of surfactin in the fructose extracts was obtained when compared to the other water miscible substrates [fructose vs glucose (p < 0.0001), fructose vs glycerol (p < 0.0001) and fructose vs sucrose (p < 0.0001)].

### Effect of carbon source on the rhamnolipid production by *Pseudomonas aeruginosa* ST5

The small-scale high throughput method (96 deep-well micro-cultures) was also utilised to culture the ST5 strain in MSM supplemented with different water immiscible (diesel, kerosene and sunflower oil) and water miscible (glycerol, glucose, fructose and sucrose) substrates (Vosloo et al. 2013). The extracts were obtained from the ST5 MSM cultures in the 96 deep-well plates by the solvent (acetonitrile) extraction method and were subjected to UPLC–MS analysis. For all the ST5 extracts (obtained from MSM supplemented with different substrates), the ion spectra in positive mode showed the main groups of molecular ions at *m/z* 477.31, 505.34, 533.37, 623.37, 651.4 and 679.43, which corresponded to the protonated [M+H]^+^ molecular species of known rhamnolipids (Table 3; Additional file 1: Figure S3). Corresponding sodium adduct [M+Na]^+^ molecular ions were also observed at *m/z* 499.29, 645.35, 527.32, 673.38, 555.35 and 701.41. The singly charged protonated [M+H]^+^ molecular species differed by a mass of 21.99 amu with the singly charged sodiated [M+Na]^+^ species of the rhamnolipids (Additional file 1: Figure S3). This was consistent in all the ST5 extracts, as well as in the rhamnolipid standard (Table 3).

**Table 3:** Summary of the rhamnolipids extracted from cultures of *P. aeruginosa* ST5, as detected with high resolution mass spectrometry (<10 ppm)

Rhamnolipid group (Abbr)	UPLC Rt (min)^a^	Proposed structures of rhamnolipids	Mono-isotopic Exp/Theor *M*_r_	Protonated species Exp/Theor *m/z*	Sodiated species Exp/Theor *m/z*	Sodiated dimeric species Exp/Theor *m/z*
mRL1	7.46	Rha–C_8_–C_10_	476.3047	477.3089	499.2896	975.5889
		Rha–C_10_–C_8_	476.2985	477.3063	499.2883	975.5868
dRL1	6.6	Rha–Rha–C_8_–C_10_ Rha–Rha–C_10_–C_8_	622.3576	623.3654	645.3471	1267.7074
	6.5		622.3564	623.3642	645.3462	1267.7026
mRL2	9.03	Rha–C_10_–C_10_	504.3305	505.3383	527.3201	1031.6501
			504.3298	505.3376	527.3196	1031.6494
dRL2	7.69, 7.85, 8.07, 8.25, 8.42	Rha–Rha–C_10_–C_10_	650.3894	651.3972	673.3772	1323.7701
			650.3877	651.3955	673.3775	1323.7652
mRL3	10.56	Rha–C_12_–C_10_	532.3640	533.3700	555.3546	1087.7201
		Rha–C_10_–C_12_	532.3611	533.3689	555.3509	1087.7120
dRL3	9.6	Rha–Rha–C_12_–C_10_	678.4177	679.4285	701.4114	1379.8352
	9.7	Rha–Rha–C_10_–C_12_	678.4190	679.4268	701.4088	1379.8278

The proposed chemical structures, theoretical (Theor) and experimental (Exp) *M*_r_ and monoisotopic *m/z* values, as well as observed UPLC retention times for representative examples are provided

^a^UPLC Retention time of main peaks corresponding to the group’s *m/z* value

**Fig. 3:**
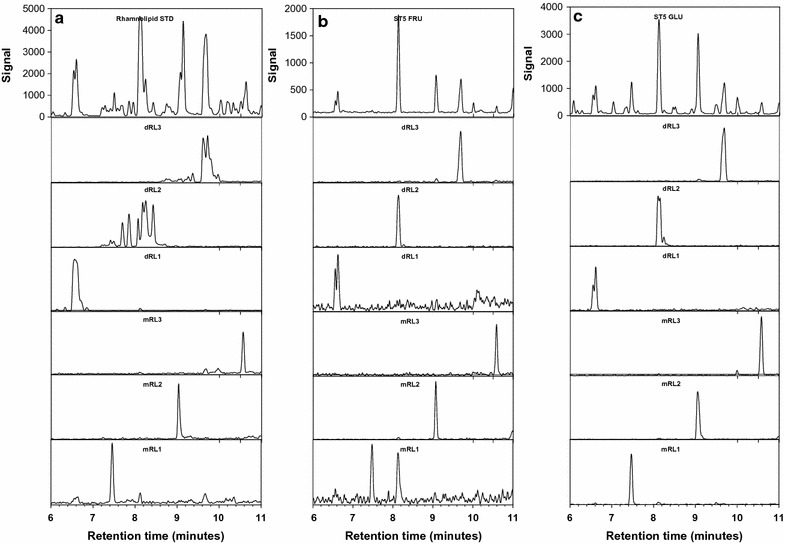
UPLC–MS profiles of rhamnolipid standard (**a**); ST5 Fructose-MSM extract (**b**); ST5 Glucose-MSM extract (**c**). The *top row *profiles show the signal of positive molecular ions detected between 6 and 11 min. Note the difference in* Y axis* which is a direct indication of amounts. The profiles below *each top row* spectrum show the extracted spectra of the rhamnolipid group

The rhamnolipid congeners detected in the culture extracts were also present in the rhamnolipid standard, which previously facilitated the identification of the congeners produced by the ST5 strain when grown in MSM supplemented with glycerol (Ndlovu 2017). Examples of the UPLC–MS profiles of the ST5 extracts from supplemented cultures are shown in Fig. 3. The ST5 extracts showed the most dominant singly charged sodiated [M+Na]^+^ molecular species at *m/z* 645.35, 673.38, 701.41, 499.29, 527.32, 555.35, which is in agreement with that of the dirhamnolipids Rha–Rha–C_8_–C_10_/Rha–Rha–C_10_–C_8_ (dRL1), Rha–Rha–C_10_–C_10_ (dRL2), and Rha–Rha–C_12_–C_10_/Rha–Rha–C_10_–C_12_ (dRL3) and monorhamnolipids, Rha–C_8_–C_10_/Rha–C_10_–C_8_ (mRL1), Rha–C_10_–C_10_ (mRL2) and Rha–C_12_–C_10_/Rha–C_10_–C_12_ (mRL3), respectively (Table 3). Extracts obtained from the ST5 strain grown in MSM supplemented with water miscible substrates (glucose, glycerol and fructose) produced six major peaks observed on the UPLC–MS profile (Fig. 3; Table 3). The sucrose MSM extract however, only produced five major peaks, which corresponded to dRL1–3 and mRL2 and 3. In comparison, the extracts obtained from the ST5 strain grown in MSM supplemented with diesel, kerosene and sunflower MSM extracts produced two (dRL2 and mRL2), five (dRL1–dRL3 and mRL1 and mRL2) and six (all rhamnolipid groups) peaks, respectively (results not shown).

The UPLC–MS profiles of the rhamnolipid standard and the extracts produced by ST5 showed significant peaks at retention times between 6 and 11 min (Fig. 3) and correlated with results obtained as outlined in Ndlovu (2017). In this study, the rhamnolipid groups eluted as follows, dirhamnolipid group 1 (dRL1) (R_t_ 6.6 and 6.5 min), 2 (dRL2) (R_t_ 7.69, 7.85, 8.07, 8.25 and 8.42 min), 3 (dRL3) (R_t_ 9.6 and 9.7 min) and monorhamnolipid group 1 (mRL1) (R_t_ 7.46 min), 2 (mRL2) (9.03 min) and 3 (mRL3) (R_t_ 10.56) (Fig. 3; Table 3; Additional file 1: Figure S3).

### Relative quantification of rhamnolipid groups in ST5 extracts

**Fig. 4:**
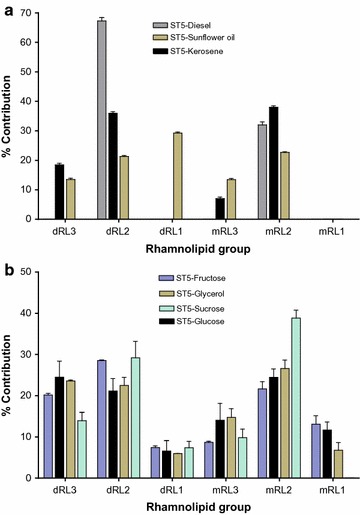
Comparison of the extracts obtained from ST5 cultivated in mineral salt medium supplemented with **a** water immiscible substrates and **b** water miscible substrates, showing the relative contribution of each of the rhamnolipid groups in the biosurfactant extracts. The contribution was calculated from UPLC profiles, with the assumption that all the rhamnolipid species have similar ion response

The approximate yields of the rhamnolipid compounds in the solvent extracts obtained from the ST5 cultures were determined using the rhamnolipid standard. This was achieved by totalling the ionisation intensities of all the sodiated [M+Na]^+^ rhamnolipid groups (dRL1–3 and mRL1–3) detected in the standard rhamnolipid, which was assumed as 1.00 mg mL^−1^ for comparative purposes only, as the absolute purity of the rhamnolipid standard is unknown. The relative ionisation intensity of each rhamnolipid group in the standard rhamnolipid was then utilised to determine the concentration of their respective individual rhamnolipid group detected in the ST5 extracts (Table 4). The approximate concentration of the total rhamnolipids produced by the ST5 strain grown in the water immiscible substrates ranged from 56 ± 49 (diesel-MSM extract) to 119 ± 37 mg L^−1^ (sunflower oil-MSM extract) (Table 4). The sunflower-MSM extract contained all six rhamnolipid groups, with dRL1 produced at 35 ± 0.5 mg L^−1^, which corresponded to a relative abundance of 29% (Fig. 4a). In contrast, the other two water immiscible extracts (diesel and kerosene) predominantly produced the dRL2 and mRL2 rhamnolipid groups, as indicated in Fig. 4a and Table 4.

**Table 4:** Comparison of the approximate quantities of each rhamnolipid group and the total rhamnolipid production profile by *P. aeruginosa* ST5 grown in mineral salt medium supplemented with different substrates as sole carbon sources

Carbon substrate	Rhamnolipid groups (mg L^−1^)						Total^a^ rhamnolipid mg L^−1^ culture
	dRL3	dRL2	dRL1	mRL3	mRL2	mRL1	
Diesel	0	38 ± 34	0	0	18 ± 15	0	56 ± 49
Kerosene	19 ± 1.2	38 ± 9.7	0	7.4 ± 13	40 ± 3.3	0.01 ± 0.0	104 ± 6.8
Sunflower oil	16 ± 3.2	25 ± 3.6	35 ± 0.5	16 ± 13	27 ± 4.3	0.5 ± 0.5	119 ± 37
Fructose	40 ± 9.5	57 ± 6.7	15 ± 26	17 ± 15	43 ± 8.7	26 ± 4.4	199 ± 57
Glucose	68 ± 32	66 ± 25	20 ± 34	43 ± 18	75 ± 34	36 ± 21	307 ± 147
Glycerol	63 ± 49	60 ± 38	16 ± 28	39 ± 37	71 ± 40	18 ± 16	267 ± 202
Sucrose	10 ± 9.9	21 ± 6.7	5.8 ± 10	7.1 ± 12	28 ± 12	0	72 ± 50
RL Standard	224.4	176	99.2	316.8	142.9	40.7	1000^a^

Each value represents the average of three culture extracts with standard error of the mean (SEM)

^a^Approximate values relative to detected signal in 1.00 mg L^−1^ rhamnolipid standard

For the water miscible substrates (glycerol, glucose, fructose and sucrose), the ST5 strain produced the highest total rhamnolipid of 307 ± 147 mg L^−1^ in the glucose-MSM extract, while the lowest concentration of 72 ± 50 mg L^−1^ was observed in the sucrose-MSM extract (Table 4). The abundance of each rhamnolipid group in the various ST5 extracts also varied, with the dRL2 and mRL2 groups constituting above 21% relative abundance each. The highest relative abundance of the mRL2 (39%) was observed in the sucrose-MSM extract however, the mRL1 group was not detected in this extract. The dRL1 and mRL1 groups were the least abundant and they were observed at <8 and 12%, respectively, in the total rhamnolipids produced by the ST5 strain (Fig. 4b). Overall, the dRL2 and mRL2 were the dominant rhamnolipid groups produced in water miscible extracts as indicated in Fig. 4b.

Statistical analysis was performed to determine if there was any significant difference between the rhamnolipid yields when ST5 was grown in MSM supplemented with different substrates. ANOVA analysis then indicated that no significant difference was observed between the rhamnolipid yields produced by the ST5 cultivated in MSM supplemented with water immiscible substrates [diesel vs kerosene (p = 0.0997), diesel vs sunflower (p > 0.0991) and kerosene vs sunflower oil (p = 0.998)]. ANOVA analysis also indicated no significant difference in the total rhamnolipid produced by ST5 grown in MSM supplemented with certain water miscible substrates [glucose vs glycerol (p = 0.9552) and fructose vs glycerol (p = 0.6461)]. However, as sucrose yielded the lowest concentration of rhamnolipid overall, a significant difference in the quantity of rhamnolipid produced in the sucrose extracts was thus obtained when compared to the other water miscible substrates [fructose vs sucrose (p = 0.0407), fructose vs glucose (p = 0.01269), glucose vs sucrose (p < 0.0001) and glycerol vs sucrose (p = 0.0002)].

## Discussion

The biosynthesis of biosurfactant compounds (glycolipids and lipopeptides) occurs on water immiscible and miscible substrates by de novo pathways, which vary in different microbial strains. Many bacterial strains produce a mixture of biosurfactant analogues and congeners, which are also influenced by the type of substrate used as a sole carbon source in the growth media (Sen 1997). In a previous study, it was indicated that the two bacterial strains ST34 (*B. amyloliquefaciens*) and ST5 (*P. aeruginosa*), isolated from wastewater, carry the *sfp* and *rhl* genes involved in the biosynthesis of surfactin and rhamnolipid, respectively (Ndlovu et al. 2016). The ST34 and ST5 strains were then confirmed to produce (extracellularly) various surfactin groups (Srf1–5) and rhamnolipid congeners, respectively, when grown in MSM supplemented with glycerol (Ndlovu 2017). Further analysis indicated that the ST34 produced five surfactin groups (Srf1–5) that were assigned to various surfactin analogues, while the ST5 produced the dirhamnolipids (Rha–Rha–C_10_–C_10_ and Rha–Rha–C_8_–C_10_/Rha–C_10_–C_8_) and monorhamnolipids (Rha–C_10_–C_10_ and Rha–C_8_–C_10_/Rha–C_10_–C_8_), as detected using a UPLC–MS methodology (Ndlovu 2017).

The solvent extracts obtained from deep-well micro-cultures of the *B. amyloliquefaciens* ST34 grown in MSM supplemented with different substrates confirmed the extracellular production of four surfactin groups (Srf1–4) (Table 1; Fig. 1). All water immiscible carbon substrates (diesel, kerosene and sunflower oil) were utilised by the ST34 strain and while four major peaks were observed, only three major surfactin groups Srf2–4 were produced, which corresponded to the C_13_–C_15_ surfactin analogues. The ST34 strain yielded a higher relative abundance of the Srf4 group containing the analogues with the longer C_15_ fatty acyl residue when grown in kerosene and sunflower MSM, with a lower abundance observed for the diesel-MSM extract. This could be because longer chain reduced carbons that were available in the substrates as precursors for longer branched fatty acyl residues. Conversely, the Srf1 group with the shortest fatty acyl residue was detected at the lowest relative abundance in the three water immiscible MSM extracts, however, the diesel MSM extract yielded slightly higher quantities of the Srf1 group compared to the sunflower and kerosene MSM extracts. In a previous study conducted by Khondee et al. (2015) a vegetable oil (palm oil) was utilised to produce a lipopeptide biosurfactant by a *Bacillus* sp. GY19. This was one of the first studies to use water immiscible substrates to increase lipopeptide production by a *Bacillus* strain and the authors indicated that an increase in the concentration of the lipopeptide was obtained when the waste glycerol together with the palm oil were used in the fermentation production process (Khondee et al. 2015).

Supplementation of the MSM with water miscible substrates for the ST34 strain again produced the four surfactin groups (Srf1–4). However, in all substrates, three major surfactin groups (Srf2–4 corresponding to C_13_–C_15_ surfactin analogues) displayed a higher abundance in the total surfactin mixture. The sucrose-MSM extract produced the highest relative abundance of the Srf3 surfactin group, while the fructose-MSM extract yielded the highest abundance of the Srf4 group. In comparison, the glycerol-MSM extract yielded the highest abundance of the Srf2 group. The lipopeptide group with the shortest fatty acid tail, Srf1, was also the least abundant in the ST34 extracts supplemented with water miscible substrates. However, the glucose-MSM extracts produced slightly higher relative abundance of the Srf1 group than those supplemented with the other water miscible carbon substrates. This result confirms that the growth medium influences the type as well as the various analogues of the biosurfactant produced. In the current study, it was however, noted that the water miscible substrates produced comparable yields of surfactin to the water immiscible substrates, with the exception of the fructose-MSM extract that yielded significantly higher quantities of total surfactin (28 ± 16 mg L^−1^). A previous study by Singh et al. (2014) indicated that the carbon source has a significant influence on the type of lipopeptides produced by *B. amyloliquefaciens* AR2. The strain AR2 produced a mixture of fengycin, iturin and surfactin variants. However, the use of sucrose and glycerol as the sole carbon sources allowed for the production of specifically the Srf2 and Srf3 surfactin groups. A study conducted by Thaniyavarn et al. (2003), then indicated that *Bacillus licheniformis* grown in nutrient yeast potato dextrose medium produced five surfactin homologues as detected by LC–MS analysis. The surfactin C_12_ (Srf1), surfactin C_13_ (Srf2), surfactin C_14_ (Srf3), surfactin C_15_ (Srf4) and surfactin C_16_ (Srf5) were produced by the *B. licheniformis* F2.2. Arutchelvi et al. (2009), also utilised glucose-MSM to produce surfactin by *Bacillus subtilis* YB7, with the C_13_ and C_14_ surfactin analogues (Srf2 and Srf3) primarily produced.

The solvent extracts obtained from the *P. aeruginosa* ST5 grown in MSM supplemented with different substrates confirmed the extracellular production of six rhamnolipid groups (dRL1–3 and mRL1–3). All the water immiscible substrates (diesel, kerosene and sunflower oil) were utilised by the ST5 strain as a sole carbon source and produced two major rhamnolipid groups dRL2 and mRL2 which corresponded to the Rha–Rha–C_10_–C_10_ and Rha–C_10_–C_10_ congeners, respectively. This in agreement with previous research where *P. aeruginosa* strains predominantly produced the Rha–Rha–C_10_–C_10_ and Rha–C_10_–C_10_ congeners when grown in water immiscible substrates (Déziel et al. 1999; Haba et al. 2003; Raza et al. 2009; Saikia et al. 2014). The ST5 strain then produced the highest relative abundance of the mRL2 group when grown in diesel MSM, with the highest abundance of the dRL2 group observed in the kerosene MSM extract. It should be noted that the diesel MSM extract only produced dRL2 and mRL2, while the six groups of rhamnolipid were detected in the sunflower oil MSM extracts.

Supplementation of the MSM with water miscible substrates also yielded all six rhamnolipid groups (dRL1–3 and mRL1–3) by the ST5 strain. However, in all miscible substrate extracts, two major rhamnolipid groups (dRL2 and mRL2) displayed relative higher abundance in the total rhamnolipid mixture as shown by the UPLC–MS data obtained for the ST5 extracts. The highest total rhamnolipid produced by ST5 strain was observed in the glucose-MSM extract (307 ± 147 mg L^−1^), followed by the glycerol-MSM extract (267 ± 202 mg L^−1^). Glycerol is the substrate most widely utilised for rhamnolipid production by *P. aeruginosa* strains (Rahman et al. 2002; Price et al. 2009; Rooney et al. 2009; Samadi et al. 2012; Rudden et al. 2015), however, results obtained in the current study indicate that glycerol and glucose produced the same rhamnolipid congeners at approximately similar concentrations. This is in agreement with a study conducted by Rudden et al. (2015), where they indicated a similar trend in rhamnolipid congeners produced by the *P. aeruginosa* strain when grown in glycerol and glucose. Furthermore, the 3-(3-hydroxyalkanoyloxy) alkanoic acids (HAAs) (C_10_–C_12_/C_12_–C_10_, C_10_–C_8_/C_8_–C_10_ and C_10_–C_10_) were detected in the ST5 extracts, as these compounds are precursors for the synthesis of Rha–Rha–C_10_–C_12_/Rha–Rha–C_10_–C_12_, Rha–C_10_–C_12_/Rha–C_12_–C_10_, Rha–Rha–C_10_–C_8_/Rha–Rha–C_8_–C_10_, Rha–C_10_–C_8_/Rha–Rha–C_8_–C_10_, Rha–Rha–C_10_–C_10_ and Rha–C_10_–C_10_, respectively. A previous study by Müller and Hausmann (2011) then indicated that the distribution of rhamnolipid congeners is dependent on the strain and culture stage. The monorhamnolipid congeners are predominantly produced at the early stationary phase, while the dirhamnolipid are predominantly synthesised towards the end of stationary phase.

Surfactin and rhamnolipid production by *B. amyloliquefaciens* ST34 and *P. aeruginosa* ST5, respectively, is significantly influenced by the substrate used as sole carbon source. Mineral salt medium supplemented with different water immiscible (diesel, kerosene and sunflower oil) and water miscible substrates (glucose, sucrose, glycerol and fructose) not only influenced the surfactin and rhamnolipid production in the deep well micro-cultures, but also the relative abundance of each surfactin analogue and rhamnolipid congener. The results indicated that higher yields of surfactins and rhamnolipids were produced by the ST34 and ST5 strains when fructose and glucose, respectively, were utilised as the sole carbon sources. The current study thus highlights the importance of the carbon source for the production of surfactin and rhamnolipid yield as well as for the variation in the analogues and possible congeners produced by the ST34 and ST5 strains, respectively. Future studies will focus on upscaling and optimising the production of the biosurfactants, as these strains alone or in combination or their products (surfactin analogues and rhamnolipid congeners), could be applied in microbial biocontrol or in bioremediation strategies, for example in soils with petrochemical contamination.

## Additional file

**Additional file 1: Figure S1.** The ESI-MS total ion mass spectra of the surfactin standard (**a**), the solvent extracted surfactin lipopeptide produced by *B. amyloliquefaciens* ST34 when grown in mineral salt medium supplemented with diesel (**b**), kerosene (**c**) and sunflower oil (**d**). The positive mass spectrum generated with MaxEnt 3 is shown. The indicated masses are [Mr + H] and [Mr + H +Na] = *m/z* values of singly charged species. **Figure S2**. The ESI-MS total ion mass spectra of the solvent extracted surfactin lipopeptide produced by *B. amyloliquefaciens* ST34 when grown in mineral salt medium supplemented with fructose (**a**), glucose (**b**), glycerol (**c**) and sucrose (**d**). The positive mass spectrum generated with MaxEnt 3 is shown. The indicated masses are [Mr + H] and [Mr + H +Na] = *m/z* values of singly charged species. **Figure S3**. UPLC-MS ion mass spectra obtained at the chromatogram peak observed at 8.1 min for the solvent extracted rhamnolipid glycolipid produced by *P. aeruginosa* ST5 when growing in mineral salt medium supplemented with glycerol (**a**), kerosene (**b**), sunflower oil (**c**) and diesel (**d**). The positive mass spectrum generated with MaxEnt 3 is shown. The indicated masses are [Mr+H] and [Mr+Na] = *m/z* values of singly charged species.

## Abbreviations

[M+H]^+^: singly charged protonated ion specie
[M+Na]^+^: singly charged sodium adduct
amu: atomic mass unit
Da: Daltons
dRL: dirhamnolipid
ESI-MS: electrospray ionisation mass spectrometry
HAAs: 3-(3-hydroxyalkanoyloxy) alkanoic acids
HCl: hydrochloric acid
*M*_*r*_: relative molecular mass
mRL: monorhamnolipid
MSM: mineral salt medium
Rha–C_8_–C_10_: α-l-rhamnopyranosyl-β-hydroxyoctanonyl-β-hydroxydecanoate
Rha–C_10_–C_8_: α-l-rhamnopyranosyl-β-hydroxydecanoyl-β-hydroxyoctanoate
Rha–C_10_–C_10_: α-l-rhamnopyranosyl-β-hydroxydecanoyl-β-hydroxydecanoate
Rha–Rha–C_8_–C_10_: α-l-rhamnopyranosyl-α-l-rhamnopyranosyl-β-hydroxyoctanonyl-β-hydroxydecanoate
Rha–Rha–C_10_–C_8_: α-l-rhamnopyranosyl-α-l-rhamnopyranosyl-β-hydroxydecanoyl-β-hydroxyoctanoate
Rha–Rha–C_10_–C_10_: α-l-rhamnopyranosyl-α-l-rhamnopyranosyl-β-hydroxydecanoyl-β-hydroxydecanoate
RL: rhamnolipid
Srf: surfactin
UPLC–MS: ultraperformance liquid chromatography coupled with mass spectrometry
