# Supplementary material for: Variants of lipopeptides and glycolipids produced by Bacillus amyloliquefaciens and Pseudomonas aeruginosa cultured in different carbon substrates
Source: AMB Express. 2017 May 31;7:109. doi: 10.1186/s13568-017-0367-4 (PMC5451376; doi:10.1186/s13568-017-0367-4)
Supplement: Supplementary file 1 — Additional file 1: Figure S1. The ESI-MS total ion mass spectra of the surfactin standard (a), the solvent extracted surfactin lipopeptide produced by B. amyloliquefaciens ST34 when grown in mineral salt medium supplemented with diesel (b), kerosene (c) and sunflower oil (d). The positive mass spectrum generated with MaxEnt 3 is shown. The indicated masses are [Mr + H] and [Mr + H +Na] = m/z values of singly charged species. Figure S2. The ESI-MS total ion mass spectra of the solvent extracted surfactin lipopeptide produced by B. amyloliquefaciens ST34 when grown in mineral salt medium supplemented with fructose (a), glucose (b), glycerol (c) and sucrose (d). The positive mass spectrum generated with MaxEnt 3 is shown. The indicated masses are [Mr + H] and [Mr + H +Na] = m/z values of singly charged species. Figure S3. UPLC-MS ion mass spectra obtained at the chromatogram peak observed at 8.1 min for the solvent extracted rhamnolipid glycolipid produced by P. aeruginosa ST5 when growing in mineral salt medium supplemented with glycerol (a), kerosene (b), sunflower oil (c) and diesel (d). The positive mass spectrum generated with MaxEnt 3 is shown. The indicated masses are [Mr+H] and [Mr+Na] = m/z values of singly charged species. [file 13568_2017_367_MOESM1_ESM.pdf]

AMB Express
SUPPLEMENTARY MATERIAL

Variants of lipopeptides and glycolipids produced by *Bacillus amyloliquefaciens* and *Pseudomonas aeruginosa* cultured in different carbon substrates

Thando Ndlovu[1], Marina Rautenbach[2], Sehaam Khan[3] and Wesaal Khan[1*]

[1]Department of Microbiology, Faculty of Science, Stellenbosch University, Private Bag X1, Stellenbosch, 7602, South Africa.

[2]BIOPEP Peptide Group, Department of Biochemistry, Faculty of Science, Stellenbosch University, Private Bag X1, Stellenbosch,7602, South Africa.

[3]Faculty of Health and Applied Sciences, Namibia University of Science and Technology, 13 Storch Street, Private Bag 13388, Windhoek, Namibia.

*Corresponding Author: Wesaal Khan, Email: wesaal@sun.ac.za, Tel: +27 21 808 5804, Fax: +27 21 808 5846

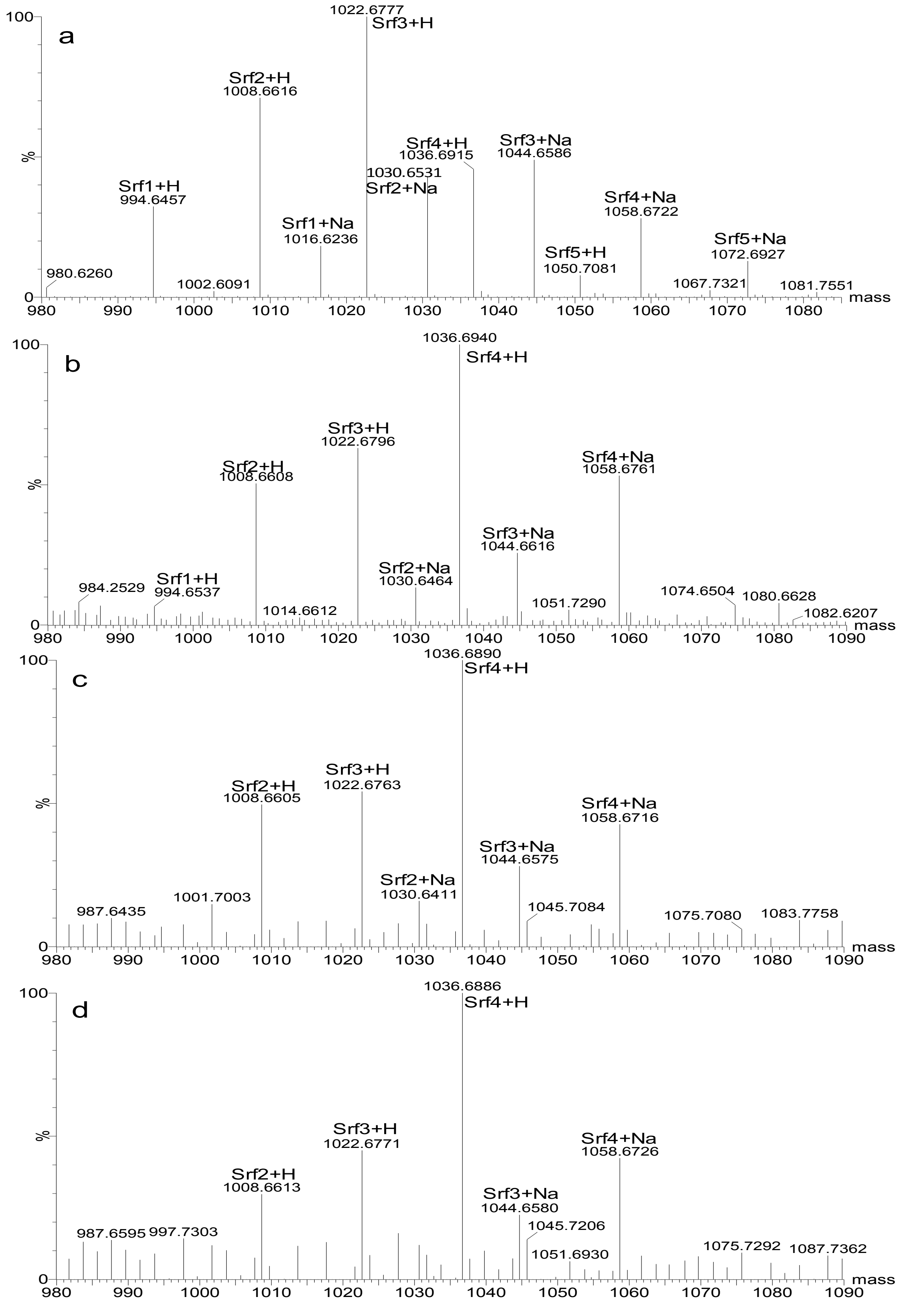

Fig. S1 The ESI-MS total ion mass spectra of the surfactin standard (a), the solvent extracted surfactin lipopeptide produced by *B. amyloliquefaciens* ST34 when grown in mineral salt medium supplemented with diesel (b), kerosene (c) and sunflower oil (d). The positive mass spectrum generated with MaxEnt 3 is shown. The indicated masses are [M_r + H] and [M_r + H +Na] = *m/z* values of singly charged species

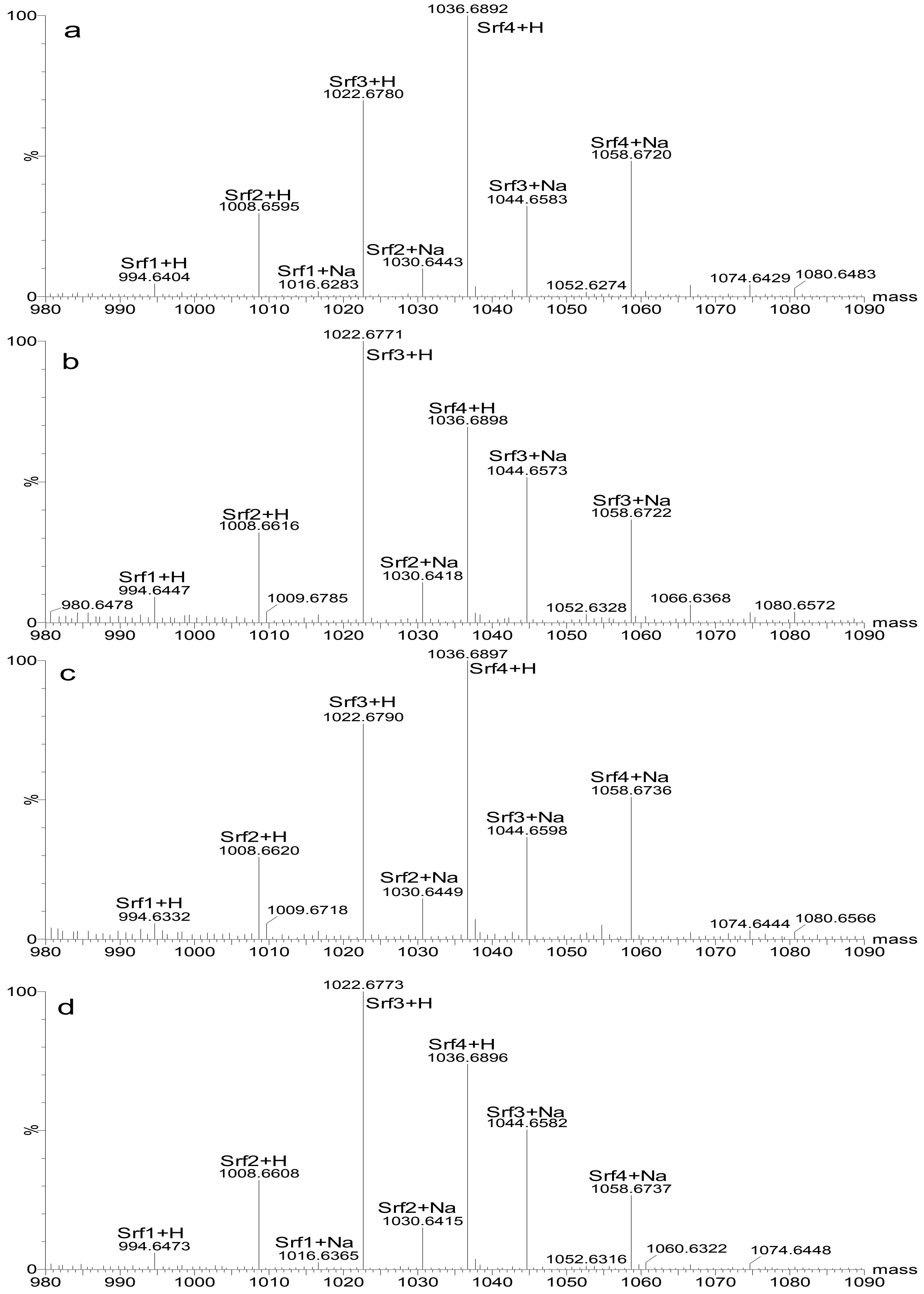

Fig. S2 The ESI-MS total ion mass spectra of the solvent extracted surfactin lipopeptide produced by *B. amyloliquefaciens* ST34 when grown in mineral salt medium supplemented with fructose (a), glucose (b), glycerol (c) and sucrose (d). The positive mass spectrum generated with MaxEnt 3 is shown. The indicated masses are [M_r + H] and [M_r + H +Na] = m/z values of singly charged species

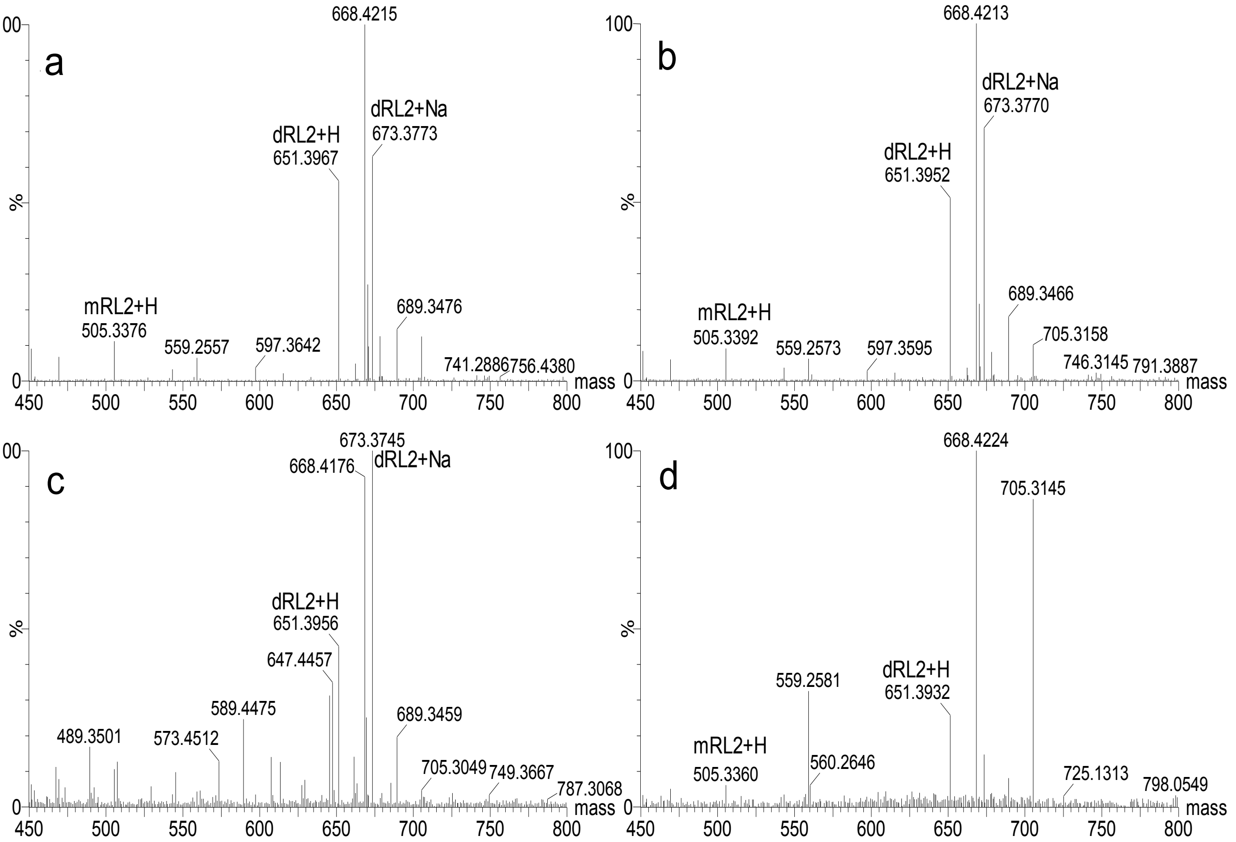

Fig. S3 UPLC-MS ion mass spectra obtained at the chromatogram peak observed at 8.1 minutes for the solvent extracted rhamnolipid glycolipid produced by *P. aeruginosa* ST5 when growing in mineral salt medium supplemented with glycerol (a), kerosene (b), sunflower oil (c) and diesel (d). The positive mass spectrum generated with MaxEnt 3 is shown. The indicated masses are [M_r+H] and [M_r+Na] = m/z values of singly charged species
